# Liver damage indices as a tool for modifying methadone maintenance treatment: a cross-sectional study

**DOI:** 10.3325/cmj.2018.59.298

**Published:** 2018-12

**Authors:** Željko Ključević, Benjamin Benzon, Nikola Ključević, Maja Veršić Bratinčević, Davorka Sutlović

**Affiliations:** 1 Public Health Institute of Split-Dalmatia County, Split, Croatia; 2 Department of Anatomy, Histology, and Embryology, University of Split School of Medicine, Split, Croatia; 3 Department of Forensic Medicine, University of Split School of Medicine, Split, Croatia; 4 Department of Toxicology and Pharmacogenetics, University of Split School of Medicine, Split, Croatia; 5 Department of Pathology, Forensic Medicine and Cytology, University Hospital Center Split, Split, Croatia

## Abstract

**Aim:**

To assess the effect of liver damage on methadone metabolism in opiate addicts undergoing methadone maintenance treatment (MMT).

**Methods:**

This cross-sectional study recruited 74 patients treated at the outpatient clinic of Public Health Institute of Split-Dalmatia County from 2013-2016. Concentrations of methadone and its main inactive metabolite were measured in participants’ biological samples on regular check-ups. Urine samples obtained before oral methadone intake, and blood and urine samples obtained 90 minutes after methadone intake were analyzed using gas chromatography/mass spectrometry. Participants were divided into groups according to liver damage criteria: hepatitis C virus status (positive, negative, or clinical remission); aspartate aminotransferase to platelet ratio (APRI) index (<0.7 and ≥0.7); and fibrosis-4 score (<1.45, 1.45-3.25, >3.25).

**Results:**

Metabolic ratio and methadone metabolite concentration in plasma decreased linearly with HCV infection status by the factor of 1.67 (*P* = 0.001) and 2.2 (*P* = 0.043), respectively. Metabolic ratio in plasma decreased in patients with APRI index ≥0.7 by the average factor of 2.12 (*P* = 0.01) and methadone metabolite concentration in plasma decreased by the factor of 6.16 (*P* = 0.009). Metabolic ratio in urine decreased with the severity of fibrosis-4 score by the average factor of 1.63 (*P* = 0.008), whereas methadone metabolite concentration decreased by the factor of 3.53 (*P* = 0.007).

**Conclusion:**

Liver damage decreases methadone metabolism. Indices of liver function should be calculated regularly during MMT for methadone dose titration.

Methadone maintenance treatment (MMT) is a common treatment approach for patients with opiate addiction (1). Physicians should individually evaluate the addiction phase and stage, together with psychological and social factors affecting the treatment course (2). Methadone dose enhancement is mainly based on patient’s subjective opinion of therapy effectiveness and clinician’s empirical judgement rather than on objective findings (3). Individual response to methadone varies because of genetic and proteomic differences as well as common psychological and mental comorbidities (4,5) that interfere with methadone metabolism by increasing toxicity and drug side effects (1). The main dose-dependent adverse effects of chronic methadone use are cardiotoxicity (1), nephrotoxicity (6), and behavioral/cognitive disorders (7,8). Therefore, clinicians should have these toxic effects in mind when optimizing MMT and determining the lowest effective dose for each individual. Furthermore, chronic addiction is a major epidemiological and social problem, especially since frequent comorbidities make the treatment challenging. Promiscuity, unprotected sexual intercourses, and drug injectors sharing (9) in this population lead to increased transmission of hepatitis C virus (HCV), hepatitis B virus, and human immunodeficiency virus (HIV) and high prevalence of chronic viral infection (10).

The liver converts methadone into an inactive metabolite, which is why it is important to monitor the liver physiological capacity during MMT. The liver’s physiological deficit could increase the amount of unmetabolized methadone and its toxic potential due to the chronic overdose (11). Although liver biopsy has been the gold standard for the diagnosis and quantification of liver damage (fibrosis), it has several disadvantages (12-14). Transient elastography, on the other hand, predicts the severity of hepatic fibrosis but not the liver metabolic capacitance (15). Cheap and easy-to-obtain alternatives to these approaches for evaluation of liver dysfunction are aspartate aminotransferase (AST) to platelet ratio (APRI) index (16) and fibrosis 4 (FIB-4) score (17), both non-invasive fibrosis indices using data from routine biochemical and hematological analyses. These tools have significant sensitivity and specificity and can be applied during each visit (16,17). FIB-4 score and APRI index have been used in a variety of models for liver damage prediction (13,18,19).

Methadone metabolism in opiate addicts undergoing MMT was studied by Wu et al, but only in patients with HCV infection (10). No study has used APRI and FIB-4 as predictors of liver damage in this group of patients and compared them with HCV infection status. Our aim was to investigate methadone metabolism properties by measuring plasma and urine concentrations of methadone and its most common inactive metabolite, 2-ethylidene-1,5-dimethyl-3,3-diphenylpyrrolidine (EDDP), and to examine if liver dysfunction influences methadone metabolism. The second aim of our study was to assess whether FIB-4 score and APRI index are useful in evaluating liver damage in patients with heroin addiction undergoing MMT treatment. Our hypothesis was that, due to disrupted liver metabolic capacity, patients with liver dysfunction taking the same initial dose of methadone as the patients with normal liver function would have greater concentration of active methadone in biological samples.

## Patients and methods

### Participants and materials

**Figure 1:**
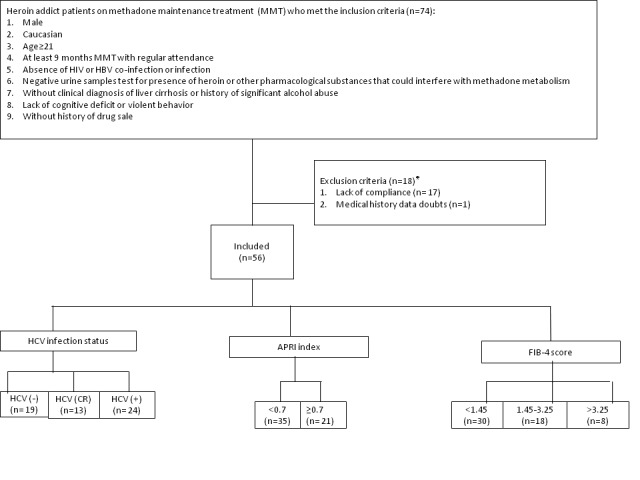
Study flowchart. HIV – human immunodeficiency virus; HBV – hepatitis B virus; CR – clinical remission; APRI – aspartate transaminase to platelet index, FIB-4 – Fibrosis-4 score. *For reasons of exclusion please see Supplementary Table 1(supplementary table 1).

This cross-sectional study was conducted in the outpatient clinic of Croatian Public Health Institute of Split-Dalmatia County between 2013 and 2016. Adult male patients with heroin addiction undergoing MMT were assessed for eligibility and 74 patients met the inclusion criteria (Figure 1). After 18 (25%) participants were excluded because of low compliance or lack of necessary data from medical records (Supplementary Table 1(supplementary table 1)), the final sample consisted of 56 participants, who were divided into groups according to their HCV status (positive, negative, or clinical remission [CR]; APRI index (<0.7 and ≥0.7); and FIB-4 score (<1.45, 1.45-3.25, >3.25) (Figure 1).

The study was conducted in accordance with the Declaration of Helsinki and approved by the Ethics Committee of the University Hospital Split, Croatia (No. 530-01/12-01/164, December 6, 2012). All participants received detailed information and signed a written informed consent for participation and data publication.

Sample size was determined on the basis of a previous study (10) and a pilot study, which included 19 adult male opiate addicts aged ≥21 years and treated within the outpatient MMT program of the Institute for Public Health of Split-Dalmatia County. Inclusion and exclusion criteria and samples collection were the same as in the present study.

### Methods

Demographic, clinical, and laboratory assessments were made at the initial meeting with the participants. The data on age, MMT dose (in milligrams), and treatment duration (in months) were obtained from medical records. Height and weight were measured as part of clinical examination, and body mass index (BMI) and weight-based dose were calculated. Interviewer-administered assessment was conducted for each participant. Aspartate aminotransferase (AST, reference range <41 IU/L), alanine transaminase (ALT, reference range <41 IU/L), and platelet count (reference range 150-400 × 10^9^/L) were determined from the blood samples analyzed at the University of Split, Department of Pathology and Forensic Medicine.

HCV status is a widely accepted predictor of liver damage (10,20). APRI index (16) and FIB-4 score (17) were used in our study as non-invasive measures for prediction of liver damage. APRI index ≥0.7 had sensitivity of 77% and specificity of 72% for predicting significant hepatic fibrosis (16). FIB-4 score <1.45 had 90% negative predictive value for the advanced fibrosis, while FIB-4 ≥ 3.25 had 97% specificity and 65% positive predictive value for advanced fibrosis (17).

Methadone and EDDP concentrations were determined in biological samples obtained from the participants at their regular check-ups. The recommended therapeutic dose for each individual was previously determined on the basis of their clinical presentation and was not modified for at least last six months. Urine samples were obtained before oral methadone administration, and blood and urine samples were obtained 90 minutes after methadone administration. The samples were stored at 4°C and analyzed within 1-4 days.

Gas chromatography/mass spectrometry drug analysis was performed using a Shimadzu GC-2010 instrument (Kyoto, Japan) as previously described (21). Briefly, racemic methadone hydrochloride and EDDP perchlorate were obtained from Lipomed AG (Arlesheim, Switzerland). Sera samples were prepared for analysis using a liquid-liquid extraction method, and 2 mL was added to pre-labeled Toxi Tubes A (Varian, Palo Alto, CA, USA) (22). The samples were extracted on the rotor and centrifuged. The supernatants were separated in glass test tubes and evaporated under a stream of inert gas of nitrogen to dryness. The dry residue was dissolved in 30 μL chloroform and transferred to a glass vial. Standard methadone and EDDP solutions were used for method validation. After EDDP and methadone plasma and urine concentration levels were measured, metabolic ratio (MR) was calculated as the concentration ratio of EDDP to methadone in urine specimens. The MR is unitless and represents the relationship of methadone to its metabolite at the time of collection (23).

### Statistical analysis

MR in urine, and MTD and EDDP concentration in plasma, were log_10_-transformed because the data followed log-normal distribution. If data are log transformed, the difference between means of data logarithms becomes a ratio, which when transformed back to the original scale becomes the ratio of two geometric means (24,25). Therefore, the effect sizes in this study are expressed as geometric mean ratios and 95% confidence intervals (CI). Significance of difference between continuous variables was analyzed using *t* test, linear test for trend, and multiple linear regression. Descriptive statistics are expressed as a median and interquartile range for continuous variables or as a percentage for proportions. Explanatory power (R^2^) represents the determination coefficient of linear models, *t* test model, or multiple linear regression. Model selection was performed using the Akaike information criterion (AIC) (26). The level of significance was set at *P* < 0.05. Study sample size was determined so that study had 80% power to detect small effect size of Cohen’s f^2^ = 0.15. Statistical analysis was performed using GraphPad Prism 7.02 software for Windows (GraphPad Software, La Jolla, CA, USA), and power calculation was performed using the G*Power software (Heinrich Heine University, Dusseldorf, Germany). Bayesian factors (BF) were calculated by using JASP software (JASP Team 2017, version 0.8.3.1, *https://jasp-stats.org*).

## Results

Twenty-four of 56 participants (43%) were HCV positive (Table 1). All participants were treated with a median methadone dose of 80 mg (range: 50-100 mg). Median duration of MMT was 90 months (range: 46-173 months), without any significant difference in dose or treatment duration between HCV-positive and HCV-negative group. APRI index higher than 0.7 was found in 27 (37.5%) participants and FIB-4 higher than 3.25 in 8 (14.28%) participants (Table 1). Median age of the entire cohort was 43 years (range: 39-50 years), and there were no significant differences between the groups in BMI or the dose relative to patients’ body weight.

**Table 1:** Demographic and laboratory findings (median and interquartile range) in study participants on methadone maintenance treatment divided according to hepatitis C virus (HCV) infection, aspartate transaminase to platelet ratio index (APRI), and fibrosis-4 score

Parameter	HCV infection			APRI		Fibrosis-4 score		
	negative (n = 19)	clinical remission (n = 13)	positive (n = 24)	<0.7 (n = 35)	≥0.7 (n = 21)	<1.45 (n = 30)	1.45-3.25 (n = 18)	˃3.25 (n = 8)
Age (years)	43 (38-52)	42 (38-49)	44 (40-49)	42 (39-49)	45 (39-50)	42 (38-48)	42 (40-49)	46 (38-54)
Treatment duration (months)	68 (49-224)	71 (53-168)	125 (37-162)	109 (43-179)	75 (70-150)	90 (48-179)	134 (43-187)	94 (51-145)
Body mass index (kg/m^2^)	24.0 (21.4-27.1)	25.0 (22.7-25.7)	26.0 (22.8-29.1)	24.0 (22.8-27.7)	26.0 (22.2-29.9)	25.0 (23.3-27.9)	26.0 (22.8-28.4)	24.0 (21.1-29.0)
Dose (mg/kg)	1.00 (0.58-1.85)	0.93 (0.38-1.07)	1.02 (0.67-1.29)	0.94 (1.25-1.92)	0.98 (0.59-1.27)	0.83 (0.55-1.22)	1.16 (0.83-1.54)	0.79 (0.37-1.04)
Aspartate transaminase (U/L)	32 (21-43)	27 (21-44)	68 (42-76)	30 (22-35)	92 (65-89)	31 (21-41)	43 (32-72)	150 (83-232)
Alanine aminotransferase (U/L)	23 (17-74)	20 (15-53)	91 (32-102)	22 (155-32)	120 (54-177)	24 (17-65)	43 (23-92)	98 (81-144)

### Chronic HCV infection and methadone metabolism

**Figure 2:**
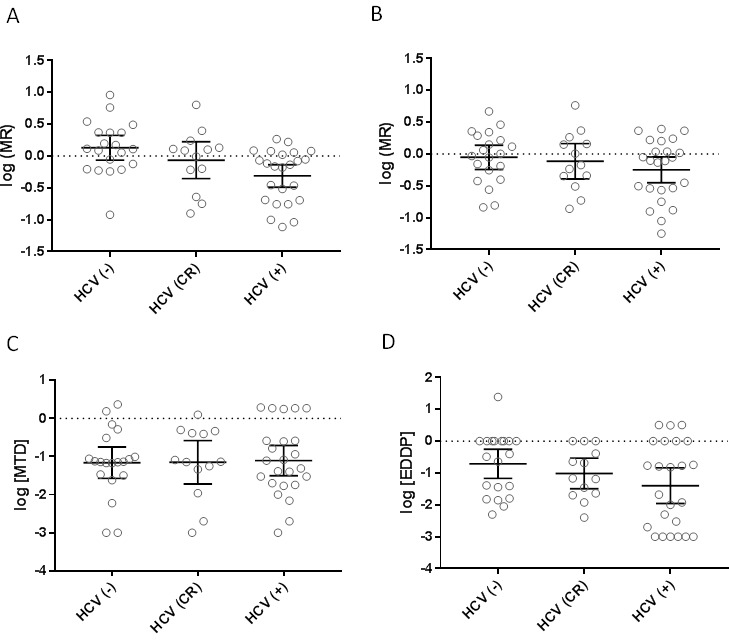
Methadone (MTD) pharmacokinetics stratified by hepatitis C virus (HCV) infection status. (**A**) A significant decrease in MR in urine samples after methadone administration (β = -0.2215, 95% CI 1.24 to 2.24, *P* = 0.001). (**B**) A non-significant difference in logarithmically transformed metabolic ratio (MR) in urine samples before methadone administration (β = -0.0994, 95% confidence interval [CI] 0.058 to 1.08, *P* = 0.153, BF = 0.7). (**C**) A non-significant difference in logarithmically transformed methadone plasma concentration (β = 0.0275, 95% CI -0.2481 to 0.3032, *P* = 0.841, BF = 0.27). (**D**) A significant difference in logarithmically transformed plasma concentration of 1,5-dimethyl-3,3-diphenylpyrrolidine (EDDP) (β = -0.3436, 95% CI 1.03 to 4.74, *P* = 0.043). β – slope parameter of log linear model, BF – Bayesian factor.

MR in urine after methadone administration decreased linearly with HCV infection status by the average factor of 1.67 (95% CI 1.24 to 2.24, *P* = 0.001; Figure 2A). A similar trend was observed before methadone administration, but the difference was not significant (fold-change in geometric mean ratio = 1.17, 95% CI 0.058-1.08, *P* = 0.153, BF = 0.7; Figure 2B). There were no differences in methadone plasma concentration in patients with different HCV infection status (Figure 2C). Plasma EDDP after methadone ingestion decreased with the HCV infection status by the average factor of 2.2 (95% CI 1.03-4.74, *P* = 0.043) (Figure 2D).

### APRI index and methadone metabolism

**Figure 3:**
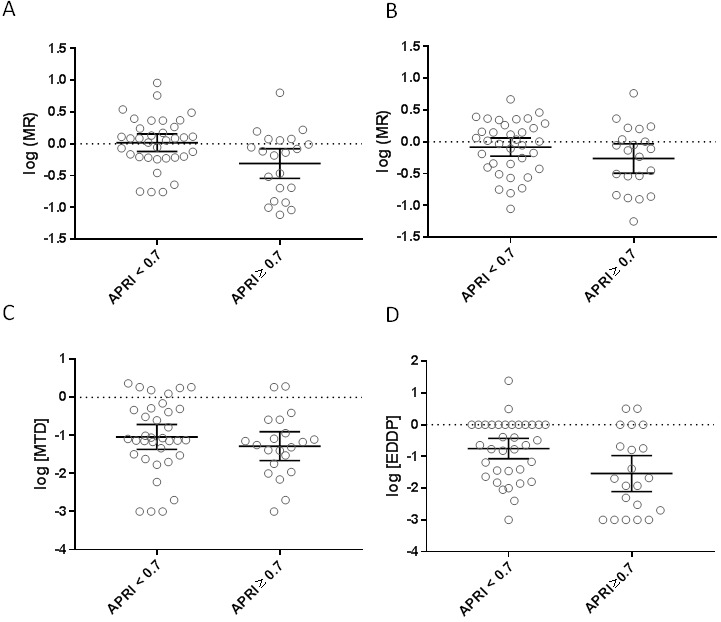
Methadone (MTD) pharmacokinetics stratified by aspartate transaminase to platelet ratio (APRI) index. A significant decrease in logarithmically transformed metabolic ratio (MR) in urine samples in patients with APRI index ≥7 compared with those with APRI index <0.7 after methadone administration (95% confidence interval [CI] 0.12 to 3.74, *P* = 0.010). (**A**). A non-significant difference in MR before methadone administration (95% CI 0.85 to 2.68, *P* = 0.154, BF = 0.65) **(B)** A non-significant difference in logarithmically transformed methadone plasma concentration according to APRI status (95% CI 0.179 to 1.819, *P* = 0.338, BF = 0.4). (**C**) A significant difference in logarithmically transformed plasma 1,5-dimethyl-3,3-diphenylpyrrolidine metabolite (EDDP) (95% CI 57 to 24, *P* = 0.009). (**D**) BF – Bayesian factor.

MR in urine after methadone administration decreased in participants with APRI index ≥0.7 by the average factor of 2.12 (95% CI 1.2- 3.74, *P* = 0.010) compared with patients with APRI index <0.7 (Figure 3A). Although a similar trend was observed before methadone administration (1.52, 95% CI 0.85-2.68, *P* = 0.154, BF = 0.65), the difference was not significant (Figure 3B). Methadone plasma concentration non-significantly increased in participants with APRI index ≥0.7 (geometric mean ratio 0.572, 95% CI 0.179 to 1.819, *P* = 0.338, BF = 0.4) (Figure 3C). Plasma EDDP concentration in patients with APRI index ≥0.7 significantly decreased by the average factor of 6.16 (95% C1.57-24, *P* = 0.009) (Figure 3D).

### FIB-4 score and methadone metabolism

**Figure 4:**
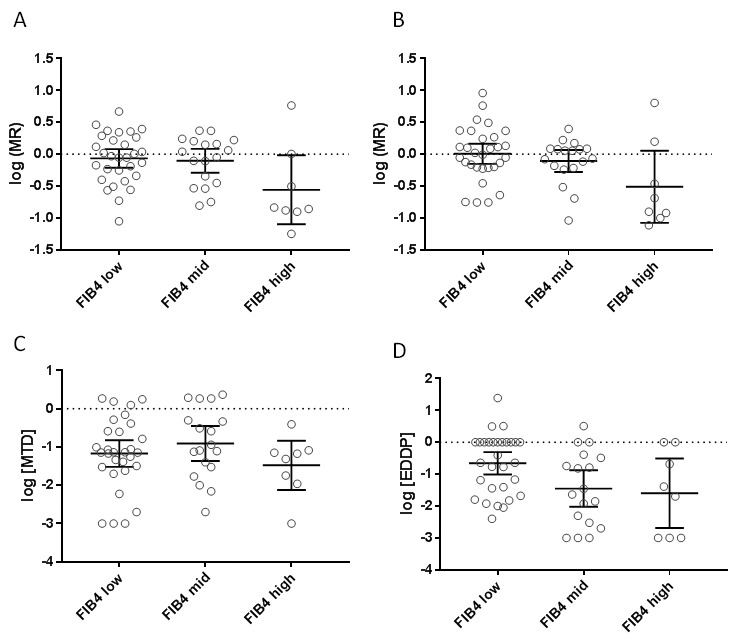
Methadone (MTD) pharmacokinetics stratified according to fibrosis-4 (FIB-4) score. A significant decrease in logarithmically transformed metabolic ratio (MR) with rising FIB-4 score before (β = -0.3567, 95% confidence interval [CI] 1.08 to 2.27, *P* = 0.018) (**A**) and after methadone administration (β = -0.2236, 95% CI 1.15 to 2.44, *P* = 0.008) (**B**). A non-significant difference in logarithmically transformed plasma methadone concentration according to FIB-4 score (β = -0.0543, *P* = 0.749, BF = 0.28) (**C**). A significant decrease in plasma 1,5-dimethyl-3,3-diphenylpyrrolidine (EDDP) in patients with high FIB-4 score (β = -0.5478, 95% CI = 1.43 to 8.72, *P* = 0.007) (**D**). β – slope parameter of log linear model, BF – Bayesian factor.

MR before and after oral methadone intake decreased with the severity of FIB-4 score by the average factor of 1.57 (95% CI 1.08 to 2.27, *P* = 0.018) and 1.63 (95% CI 1.15 to 2.44, *P* = 0.008), respectively (Figure 4A and 4B). Methadone plasma concentration was not associated with the severity of FIB-4 score (Figure 4C), whereas plasma EDDP concentration decreased with increasing FIB-4 score by the factor of 3.53 (95% CI 1.43 to 8.72, *P* = 0.007) (Figure 4D).

### Comparison of liver damage indices

To determine which of the liver damage indices better explains changes in methadone metabolism (Table 2), we compared them in terms of explanatory power (R^2^) for differences in methadone and EDDP plasma concentration and MR in urine. Overall, the best explanatory power was shown by FIB-4 score. R^2^ for stratification of methadone plasma concentration of FIB-4 was two times higher than that for APRI and 6 times higher than that for HCV infection status. For EDDP plasma concentration, non-invasive fibrosis indices offered better explanatory power than the HCV infection status (Table 2). For MR determination in urine, the best stratification variable was HCV infection status, although the explanatory power of all liver damage indices remained rather low. All three predictors better explained differences in urine MR and plasma EDDP than plasma methadone. The optimal model for predicting methadone metabolism based on HCV, FIB4, and other demographic and morphometric characteristics of our participants (Supplementary Table 2(supplementary table 2)) predicted log of MR after methadone intake based on HCV status, FIB-4, and participant age, with an explanatory power of 27% (Supplementary Table 3(supplementary table 3)).

**Table 2:** Explanatory power (R^2^) of stratification variables of liver damage predictors including hepatitis C virus (HCV) infection status, aspartate transaminase to platelet ratio index (APRI), and fibrosis-4 score for difference in plasma methadone, plasma 2- ethylidene-1,5-dimethyl-3,3-diphenylpyrroldine (EDDP), and urine metabolic ratio

Main outcome measures	Stratification variables		
	HCV status	APRI	fibrosis-4 score
**Plasma methadone (mg/L, %)**	0.7	2.1	4.2
**Plasma EDDP (mg/L, %)**	7.3	11.7	12.6
**Urine metabolic ratio (%)**	17.7	11.6	13.9

## Discussion

We found a significant association between non-invasive predictors of liver damage and a decrease in urine and plasma EDDP concentration, probably due to impaired liver methadone metabolism. MR changes could be attributed to lower overall EDDP concentration rather than higher methadone concentration, which indicates diminished liver capacity for methadone processing.

There was no difference in plasma methadone levels between participants with different HCV infection status, FIB-4 score, or APRI index. This could be explained by methadone’s lipophilic properties and high volume of distribution (27). Methadone concentration in tissues is higher than in plasma, with only about 2% of absorbed methadone remaining in the plasma compartment (27). Therefore, it is understandable that we could not find a difference in the plasma methadone concentration between the groups; plasma methadone represents a small fraction of the total methadone concentration following oral administration.

Our results do not confirm previous findings (20) of higher initial methadone doses in HCV-positive patients. In our study, the initial methadone dose was around 1 mg/kg regardless of BMI, treatment duration, HCV infection status, or participant’s age and did not differ between the groups. However, it seems that this dose if orally administered does not provide the same drug amount or concentration to all patients. This could be explained by different pharmacokinetic profiles in patients with APRI≥0.7 index and FIB-4 score >3.25, hence depending on an individual hepatic capacity for methadone metabolism.

Although MR in urine, as an overall measure of methadone metabolic capacity, and EDDP plasma concentration decreased linearly with HCV infection status, we did not observe any differences in methadone plasma concentration. These findings differ from the findings by Wu et al (10), who reported higher plasma methadone concentration in patients with HCV infection, together with lower MR. These discrepancies may be attributed to differences in race, sample size, or inclusion criteria. Unlike Wu et al (10), we divided the participants according to HCV status, distinguishing HCV-positive from those in remission and excluded patients co-infected with hepatitis B virus or HIV. Finally, the difference could be attributed to the surprisingly high rate of around 95% HCV-positive patients in the study by Wu et al (10), compared with 43% in our study.

Furthermore, in our study, patients with APRI index ≥0.7 and FIB-4 score 1.45-3.25 and >3.25 had decreased MR in urine and EDDP plasma concentration. Although there are several reports on the usefulness of FIB-4 score and APRI index in a variety of models for liver damage prediction (13,19), this is, to the best of our knowledge, the first report evaluating the use of FIB-4 score and APRI index in methadone metabolism prediction and dose modification in heroin abusers undergoing MMT treatment.

The best explanatory power was obtained by FIB-4 score. APRI index had three times higher explanatory power for plasma methadone when compared with HCV infection status. These results suggest that indices of liver fibrosis better assess methadone metabolism in patients undergoing MMT than clinical diagnosis of HCV infection status.

One of the study limitations is the small sample size, but the poor compliance and motivation of these patients should be taken into consideration when conducting this type of studies. Another limitation is the lack of female participants, who were not recruited due to small number of women treated at our institution. Blood samples before methadone administration were not collected because IV drug users often suffer from damaged blood vessels, which makes the sampling difficult and sometimes impossible. Furthermore, the blood methadone concentration reaches the peak value of 2.5 about 4 h after therapeutic dose administration, during which time participants become less compliant (28). These blood samples could have provided more information on liver metabolic capacity and methadone body elimination. Additionally, this study did not consider genetic influence of CYP3A4 and CYP2B6 and molecular properties of methadone elimination, which is an issue that merits further study.

In conclusion, this is the first study evaluating the use of APRI index and FIB-4 score in methadone metabolism prediction and comparing these indices with HCV infection status. Liver fibrosis indices and HCV status were to a lesser extent associated with decreased methadone liver-mediated metabolism in patients with heroin addiction suitable for MMT program. Therefore, calculation of these indices could be a potential tool for clinicians in everyday practice. Future research should assess the correlation between the mentioned variables and methadone-dosing regimen to determine whether these variables may be used to titrate methadone target doses in patients starting methadone maintenance program.
