# Supplementary material for: Liver damage indices as a tool for modifying methadone maintenance treatment: a cross-sectional study
Source: Croat Med J. 2018 Dec;59(6):298–306. doi: 10.3325/cmj.2018.59.298 (PMC6330771; doi:10.3325/cmj.2018.59.298)
Supplement: Supplementary Table 1 [file CroatMedJ_59_s001.pdf]

Supplementary Table 1. Demographic and clinical data of patients in methadone maintenance treatment who did not meet the inclusion criteria

Patient	Reason of exclusion	Hepatitis C virus status	Age (years)	Weight (kg)	Height (cm)	Body mass index(kg/m^2)	Methadone dose (mg)	Methadone maintenance treatment duration (months)
1.	Blood samples could not be obtained	positive	45	73	171	25	75	159
2.	Had an acute psychotic episode	positive	52	87	185	25.4	100	100
3.	Did not attend the second appointment	positive	34	78	173	26.1	100	162
4.	Blood samples could not be obtained	positive	40	65	180	20.1	100	160
5.	Withdrew from experiment	positive	37	80	175	26.1	100	127
6.	Withdrew from experiment	positive	52	82	174	27.1	125	211
7.	Recalled consent	positive	45	75	171	29.1	100	274
8.	Recalled consent	positive	51	80	198	20.4	100	251
9.	Unnoticed alcohol issues	positive	44	70	185	20.5	100	211
10.	Did not attend the second appointment	positive	51	68	172	23	100	268

11.	Withdrew from experiment	positive	46	82	187	23.4	100	187
12.	Recalled consent	positive	51	90	170	31.1	100	267
13.	Did not attend the second appointment	positive	43	90	187	25.7	25	254
14.	Blood samples could not be obtained	negative	31	90	185	26.3	80	46
15.	Recalled consent	negative	26	75	179	23.4	100	57
16.	Withdrew from experiment	negative	37	95	180	29.3	90	155
17.	Withdrew from experiment	negative	45	83	190	23	125	65
18.	Withdrew from experiment	negative	34	72	175	23.5	100	35
