# Supplementary material for: Liver damage indices as a tool for modifying methadone maintenance treatment: a cross-sectional study
Source: Croat Med J. 2018 Dec;59(6):298–306. doi: 10.3325/cmj.2018.59.298 (PMC6330771; doi:10.3325/cmj.2018.59.298)
Supplement: Supplementary Table 2 [file CroatMedJ_59_s002.pdf]

Supplementary Table 2. Selection process for log linear model which predicts log of metabolic ratio in urine after methadone intake*

Variables	Akaike information criterion (corrected)	Evidence ratio	Probability that model is correct
**FIB-4, HCV, age, BMI**	-90.65	2.745601015	0.266979851
**FIB-4, HCV, age**	-92.67	1	0.5
**FIB-4, HCV**	-91.59	1.716006862	0.368187582
**FIB-4**	-86.42	22.75989509	0.042087728
**HCV**	-89.86	4.075526741	0.197023886
**HCV, age**	-89.4	5.129457997	0.163146562
**FIB-4, age**	-87.34	14.36794955	0.06507049

***FIB-4 – fibrosis-4; HCV – hepatitis C virus; BMI – body mass index.**
