# Supplementary material for: Liver damage indices as a tool for modifying methadone maintenance treatment: a cross-sectional study
Source: Croat Med J. 2018 Dec;59(6):298–306. doi: 10.3325/cmj.2018.59.298 (PMC6330771; doi:10.3325/cmj.2018.59.298)
Supplement: Supplementary Table 3 [file CroatMedJ_59_s003.pdf]

Supplementary Table 3. Coefficients of log linear model predicting log of metabolic ratio in urine after methadone intake

**Parameter estimates**	**Variable**	**Estimate**	**Standard error**	**95% confidence interval**	**P value**
**β0**	Intercept	-0.394	0.3346	-1.065 to 0.2774	0.2444
**β1**	Hepatitis C virus status	-0.1822	0.0656	-0.3138 to -0.05051	0.0076
**β2**	Age	0.01381	0.007544	-0.001333 to 0.02895	0.073
**β3**	Fibrosis-4 category	-0.1899	0.08053	-0.3515 to -0.02828	0.0222
**R^2**					**27.76%**
